# Supplementary material for: (+)-Lipoic Acid Reduces Lipotoxicity and Regulates Mitochondrial Homeostasis and Energy Balance in an In Vitro Model of Liver Steatosis
Source: Int J Mol Sci. 2023 Sep 23;24(19):14491. doi: 10.3390/ijms241914491 (PMC10572323; doi:10.3390/ijms241914491)
Supplement: Supplementary file 1 [file ijms-24-14491-s001.zip › ijms-2525105-supplementary.pdf]

International Journal of
Molecular Sciences

Article

(+)-Lipoic Acid Reduces Lipotoxicity and Regulates Mitochondrial Homeostasis and Energy Balance in an In Vitro Model of Liver Steatosis

Lucia Longhitano [1†], Alfio Distefano [1†], Angela Maria Amorini [1], Laura Orlando [1], Sebastiano Giallongo [1], Daniele Tibullo [1], Giuseppe Lazzarino [1], Anna Nicolosi [2], Amer M. Alanazi [3], Concetta Saoca [4], Vincenzo Macaione [4], M'hammed Aguennouz [4], Federico Salomone [5], Emanuela Tropea [1], Ignazio Alberto Barbagallo [1], Giovanni Li Volti [1,*] and Giacomo Lazzarino [6]

1 Department of Biomedical and Biotechnological Sciences, University of Catania, 95123 Catania, Italy; (L.L.) lucia.longhitano@unict.it; (A.D.) distalfio@gmail.com; (A.M.) amorini@unict.it; (L.O.) lauraorlando2810@gmail.com; (S.G.) sebastiano.giall@gmail.com; (D.T) d.tibullo@unict.it; (G.L.) lazzarig@unict.it; (E.T.) tropeaemanuela3@gmail.com; (I.A.B) ignazio.barbagallo@unict.it; (G.L.V.) livolti@unict.it;
2 Hospital Pharmacy Unit, Ospedale Cannizzaro, 95125 Catania, Italy; (A.N.) annanicolosi@hotmail.com;
3 Pharmaceutical Biotechnology Laboratory, Department of Pharmaceutical Chemistry, College of Pharmacy, King Saud University, Riyadh 11451, Saudi Arabia; (A.M.A) amalanazi@ksu.edu.sa;
4 Depart. Clinical and Experimental Medicine, University of Messina Via Consolare Valeria 1 98125 Messina; (M.A.) aguenoz.mhommed@unime.it; (C.S.) saoca.concetta@unime.it; (V.M.) vincenzo.macaione@unime.it
5 Division of Gastroenterology, Ospedale di Acireale, Azienda Sanitaria Provinciale di Catania, Catania, Italy; (F.S.) federicosalomone@rocketmail.com
6 UniCamillus-Saint Camillus International University of Health Sciences, Via di Sant'Alessandro 8, 00131 Rome, Italy; (G.L.V.) Giacomo.lazzarino@unicamillus.org
† These authors contribute equally to the manuscript .
* Correspondence: livolti@unict.it.

Supplementary Materials

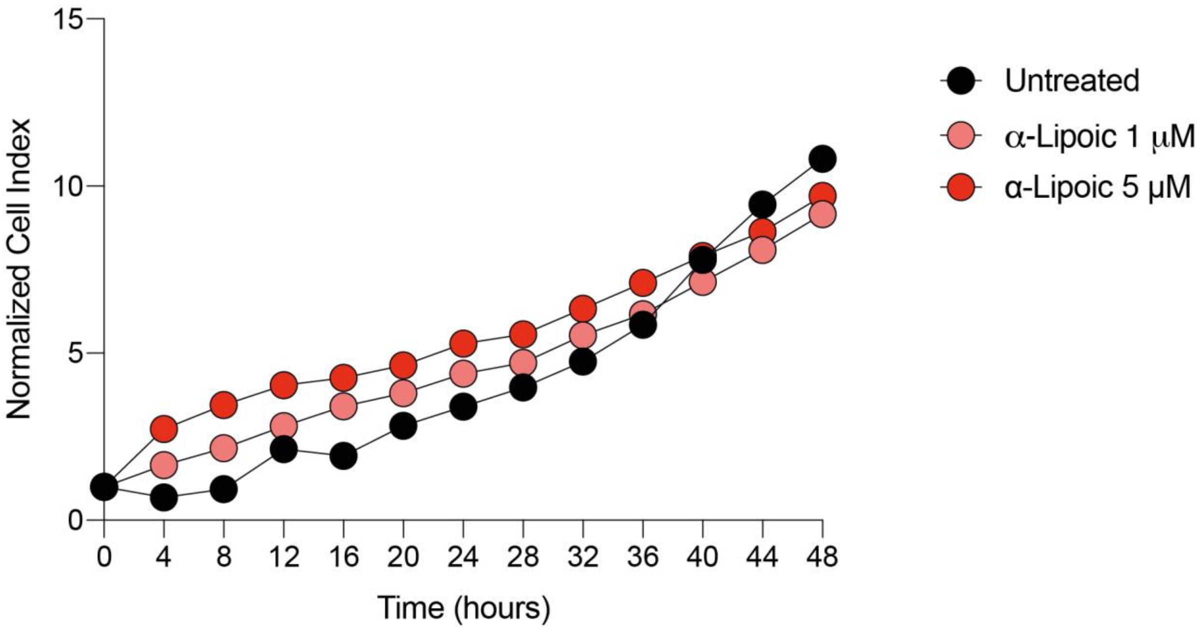

Figure S1.

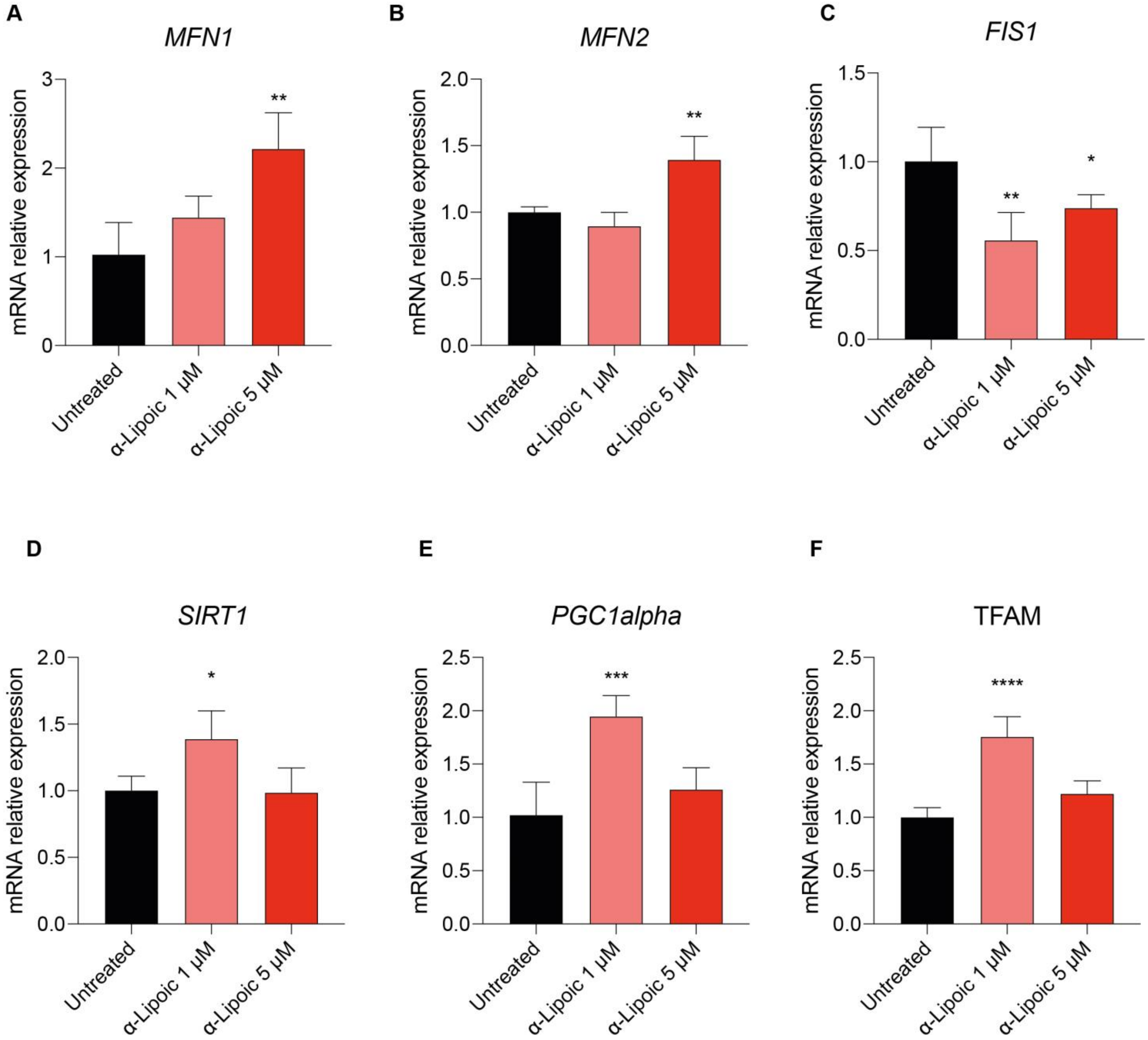
A
MFN1
mRNA relative expression
0
1
2
3
**
Untreated
α-Lipoic 1 μM
α-Lipoic 5 μM
B
MFN2
mRNA relative expression
0.0
0.5
1.0
1.5
2.0
**
Untreated
α-Lipoic 1 μM
α-Lipoic 5 μM
C
FIS1
mRNA relative expression
0.0
0.5
1.0
1.5
**
*
Untreated
α-Lipoic 1 μM
α-Lipoic 5 μM
D
SIRT1
mRNA relative expression
0.0
0.5
1.0
1.5
2.0
*
Untreated
α-Lipoic 1 μM
α-Lipoic 5 μM
E
PGC1alpha
mRNA relative expression
0.0
0.5
1.0
1.5
2.0
2.5
***
Untreated
α-Lipoic 1 μM
α-Lipoic 5 μM
F
TFAM
mRNA relative expression
0.0
0.5
1.0
1.5
2.0
2.5
****
Untreated
α-Lipoic 1 μM
α-Lipoic 5 μM

Figure S2.

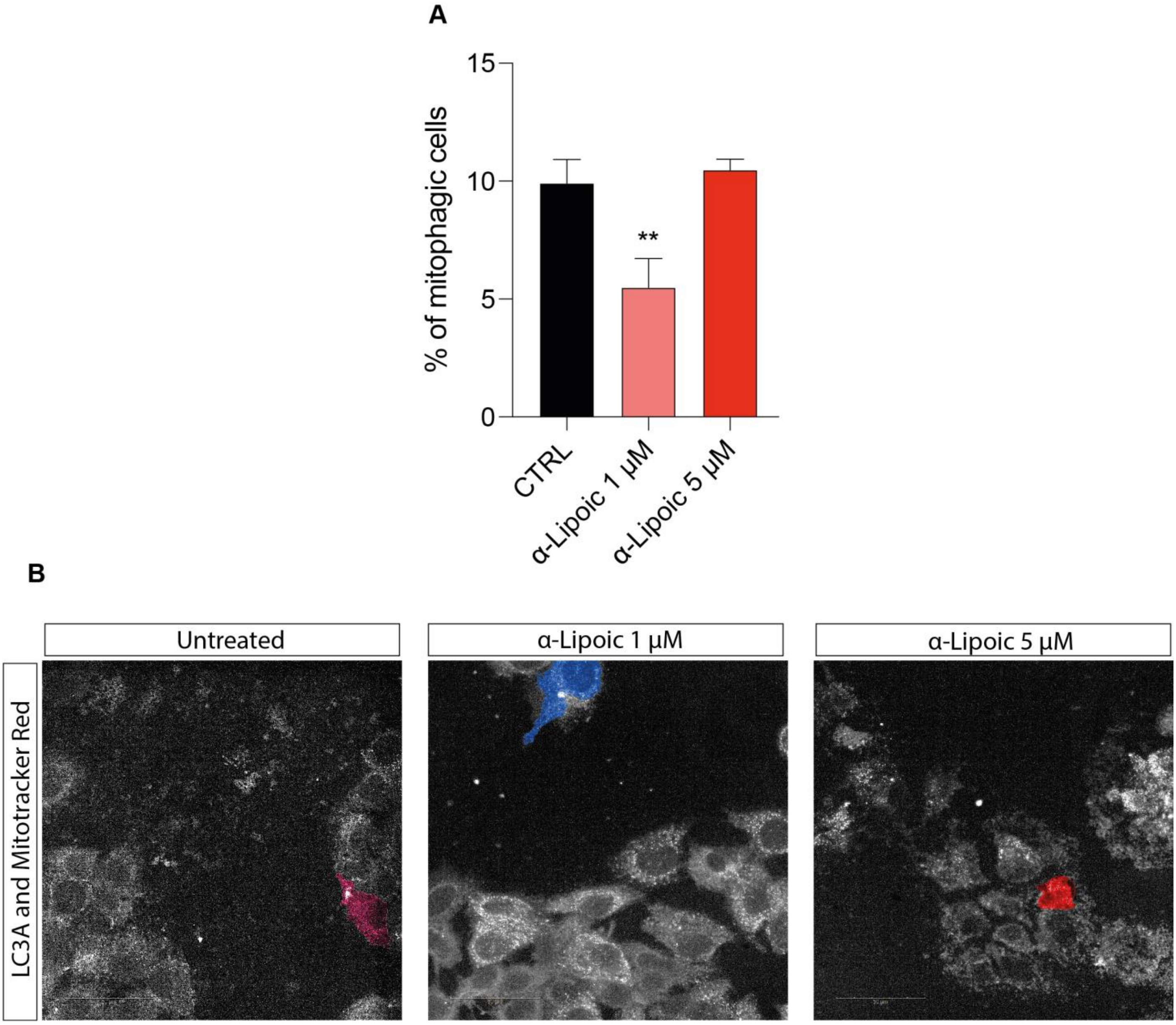
A
% of mitophagic cells
15
10
5
0
**
CTRL
α-Lipoic 1 µM
α-Lipoic 5 µM
B
LC3A and Mitotracker Red
Untreated
α-Lipoic 1 µM
α-Lipoic 5 µM

Figure S3.

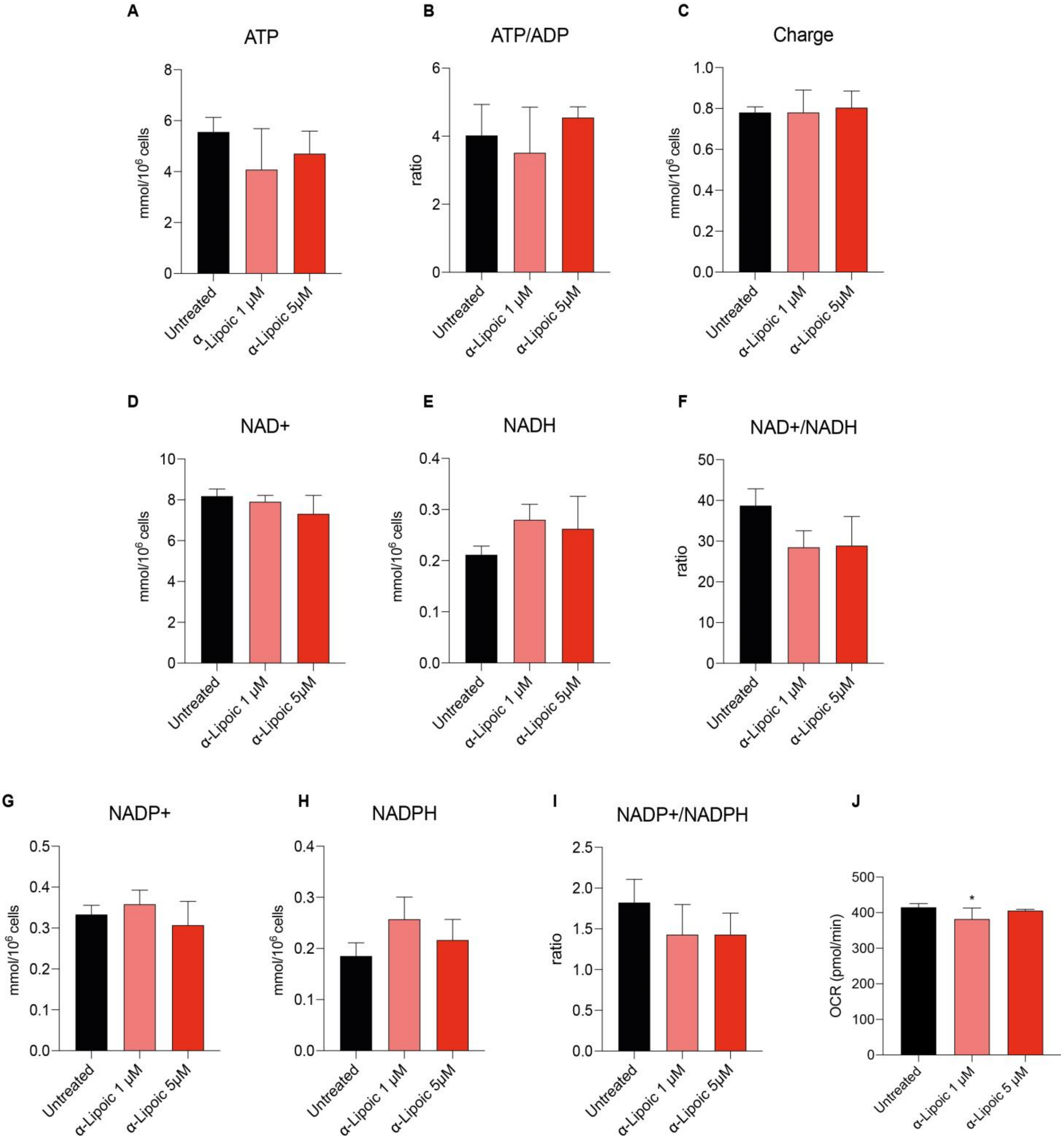
A
ATP
mmol/10^6 cells
Untreated
α-Lipoic 1 μM
α-Lipoic 5μM
B
ATP/ADP
ratio
C
Charge
D
NAD+
E
NADH
F
NAD+/NADH
G
NADP+
H
NADPH
I
NADP+/NADPH
J
OCR (pmol/min)
α-Lipoic 5 μM

Figure S4.
